# Laparoendoscopic single site adrenalectomy: initial results of cosmetic satisfaction and the potential for postoperative pain reduction

**DOI:** 10.1186/1471-2490-13-21

**Published:** 2013-04-12

**Authors:** Akira Sasaki, Hiroyuki Nitta, Koki Otsuka, Satoshi Nishizuka, Shigeaki Baba, Akira Umemura, Keisuke Koeda, Masaru Mizuno, Go Wakabayashi

**Affiliations:** 1 Department of Surgery, Iwate Medical University School of Medicine, 19-1 Uchimaru, Morioka 020-8505, Japan

**Keywords:** Laparoendoscopic single site surgery (LESS), Single-port surgery, Single-incision surgery, Laparoscopy, Adrenalectomy

## Abstract

**Background:**

Recent reports have suggested that laparoendoscopic single site (LESS) surgery is technically feasible. The aim of this study was to describe our initial experience with LESS adrenalectomy for benign adrenal tumors, focusing the attention about cosmetic satisfaction and reduction of postoperative pain.

**Methods:**

Medical records of consecutive patients undergoing LESS adrenalectomy were analyzed. All procedures were performed through a single multichannel port. Demographic and operative data were assessed. A visual analog scale (VAS) was used with a 10-point scale for an objective assessment of incisional pain and incisional cosmesis.

**Results:**

Between January 2010 and July 2012, 14 consecutive patients with benign adrenal tumors underwent LESS adrenalectomies. Of the planned LESS adrenalectomies, 12 (86%) were completed with a single-port, whereas two required an additional port placement. Mean operating time was 128.1 ± 31.5 min and mean blood loss 10.5 ± 12.1 ml. Mean pain scores using the VAS on postoperative days 1, 3, and 14 were 2.3, 1.0, and 0.3 points, respectively. The rate of analgesic use was also lower within 12 hours after surgery (14%). The patient was highly satisfied with the single small wound procedure, and mean cosmesis scores of postoperative days 3 and 14 were 9.4 and 9.8 points, respectively. The postoperative course was uneventful with no morbidity within one month of follow-up.

**Conclusions:**

LESS adrenalectomy is a safe and technically feasible procedure for patients with benign adrenal tumors, and offers cosmetic benefit and the potential for postoperative pain reduction. However, surgeons with lack of experience as LESS surgery should be comprehended that the assistance of the needlescopic instrument does not compromise the cosmetic outcomes for difficult cases and the obese patients may not always be suitable candidates for pure LESS technique.

## Background

A conventional multiport laparoscopic adrenalectomy (MPLA) using three or four ports is the gold standard operative treatment for benign adrenal tumors [1-3]. The advantages of MPLA include decreased pain, shorter hospital stay, and an earlier return to normal activity. Recently, a laparoendoscopic single site (LESS) surgery was developed as an extension of the standard laparoscopic minimally invasive procedures. LESS surgery has the potential to provide patients with improved cosmesis and decreased pain; as such, it satisfies a growing demand for less invasive surgical procedures [4,5]. Since the initial report of laparoscopic adrenalectomy using a single-incision [6], several investigators have demonstrated the technical feasibility of a variety of LESS procedures for adrenal tumors [7,8]. LESS surgery obviates the need to externally space ports for triangulation, thus allowing for the creation of a small, solitary portal of entry into the abdomen. However, we have previously reported our initial developmental experiences with select LESS procedures [9-13]. The aim of this study was to describe our initial experience with LESS adrenalectomy for benign adrenal tumors, focusing the attention about cosmetic satisfaction and reduction of postoperative pain.

## Methods

### Patients

Data were prospectively entered in an LESS adrenalectomy database and retrospectively reviewed. Between January 2010 and July 2012, 14 consecutive patients (7 men and 7 women) with benign adrenal tumors underwent LESS adrenalectomies at the Iwate Medical University Hospital. All procedures were performed by a single surgeon. Informed consent was received from all the patients for the procedure, and the difference between the LESS and the conventional multiport approaches were explained. This study was approved by the institutional review board at Iwate Medical University Hospital and conducted in accordance with Declaration of Helsinki.

As a general principle, indications for LESS adrenalectomy were hormonally active tumors ranging in size up to 5 cm, and non-functioning tumors greater than 3 cm, or with proven growth (Table 1). Exclusion criteria were suspected adrenocortical carcinoma, invasive pheochromocytoma, or high-risk patients with poor general condition.

**Table 1:** Indications for LESS adrenalectomy

**Factor**	**Feature**	**Indication**
Lesion	Size	< 5 cm
	Location	Unilateral
Patient	BMI	Non-obese
	Previous upper abdominal surgery	No
	Disease	Functioning and nonfunctioning tumors

BMI, body mass index.

A visual analog scale (VAS) was used as an objective assessment of incisional pain on postoperative days 1, 3, and 14, and for incisional cosmesis on postoperative days 3 and 14. The VAS was used to score incisional pain on a 10-point scale with a range from 0 (no pain) to 10 (worst possible pain). The VAS was also used to score cosmesis on a 10-point scale with a range from 0 (worst) to 10 (best). Data are expressed as mean ± standard deviations (SD).

### Surgical technique

**Figure 1:**
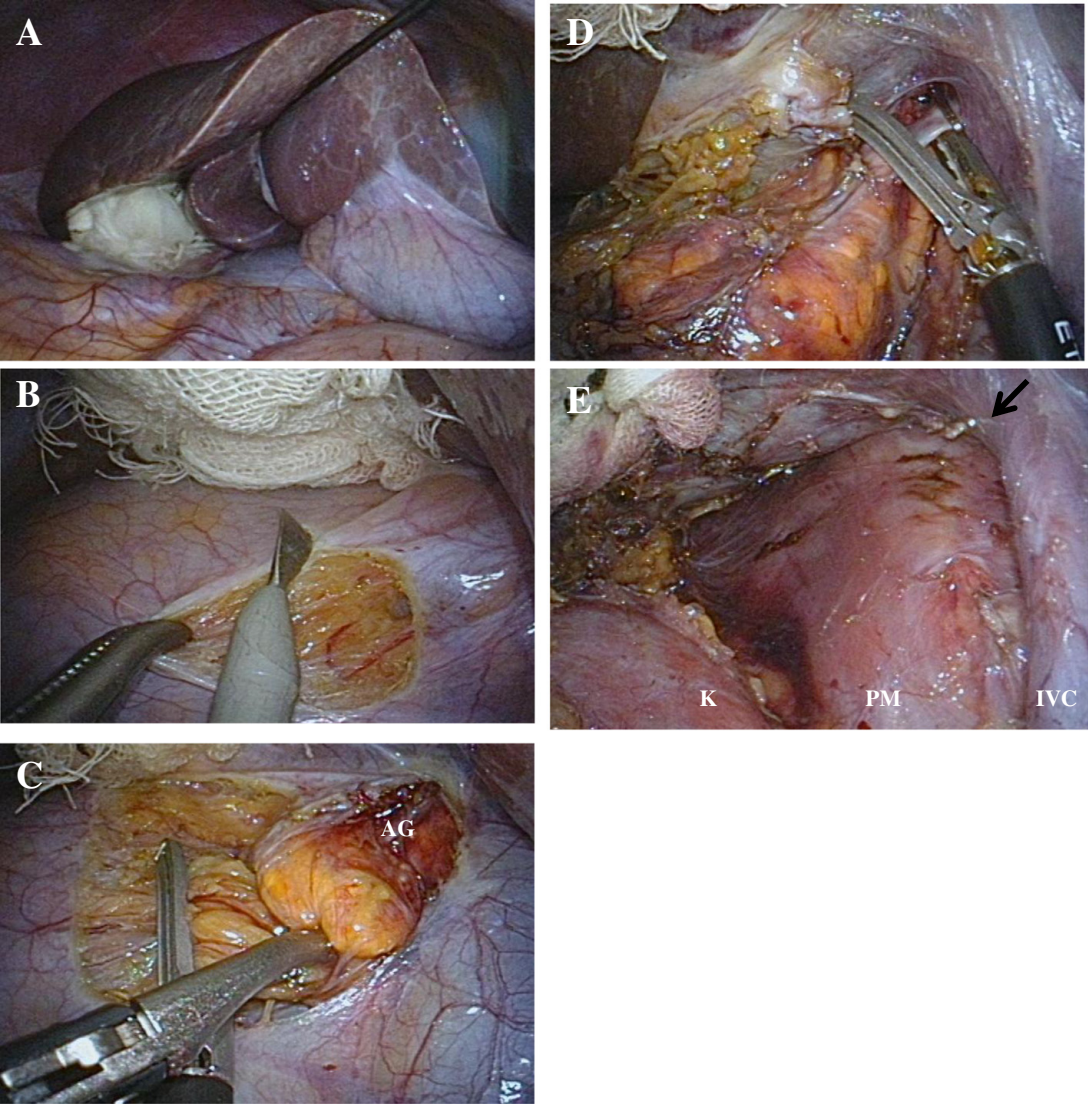
**Intraoperative view of LESS right adrenalectomy. A**: The liver was evaluated using a MiniLap and small gauze. **B**: The peritoneum and Gerota’s fascia was incised using an electrocautery. **C**: Small adrenal vessels were divided using an EnSeal. **D**: The right main adrenal vein was clipped and divided. **E**: The retroperitoneal space was examined for any evidence of bleeding. *Arrow* indicates right main adrenal vein. *AG*; adrenal gland, *K*; kidney, *PM*; psoas muscle, *IVC*; inferior vena cava.

The patient was placed in the semilateral position. All procedures were performed through a single multichannel port. A 2.5 cm incision was made through the umbilical skin and fascia. The wound protector/sleeve of the GelPOINT (Applied Medical, CA, USA) was placed. Three 5-mm cannulas were placed through the GelPOINT and then attached to the wound protector. A 5-mm flexible laparoscope (Olympus Medical Systems, Tokyo, Japan), a SILS dissector (Covidien, NewHaven, CT, USA), and a tissue sealing device (EnSeal, Ethicon, Cincinnati, OH, USA) were the primary tools used in the operation. An anterior approach, with no mobilization of the right lobe, was used for the right-sided tumors, while a lateral approach was used for left-sided tumors. Only the central adrenal vein was clipped and the small adrenal vessels were divided using an EnSeal device. For the right-sided adrenal tumor, the right liver lobe was evaluated using a 2.3 mm percutaneous instrument (MiniLap, Stryker, Kalamazoo, MI, USA) and a small gauze, which provided good visualization of the operative field surrounding the right adrenal gland. Although MiniLap insertion appears to be scar-less, a needle instrument can be traumatic for the liver. To avoid a traumatic procedure, we used small gauze as cushioning to evaluate the liver. The overall procedure was similar to the procedure performed in a conventional anterior approach using a four-port technique (Figure 1). The adrenal gland was extracted without a retrieval bag. The umbilical fascia was closed with a 2-0 Vicryl suture. The skin was closed with absorbable suture and skin glue. No drains were inserted.

## Results

Patient demographics are summarized in Table 2. Mean age was 51.7 ± 11.6 years. Indications for LESS adrenalectomy were 11 aldosterone-producing adenomas, 2 non-functioning tumors, and 1 patient with Cushing’s syndrome. Operative outcomes are detailed in Table 3. Of the planned LESS adrenalectomies, 12 (86%) were completed with a single-port, whereas two required an additional port placement for patients with body mass indexes greater than 27 kg/m^2^. Mean operating time was 128.1 ± 31.5 min and mean blood loss was 10.5 ± 12.1 ml. There was no difference between the initial 7 cases and the last 7 cases in mean operating time (134.4 min versus 121.9 min, p=0.477). There were also no other significant differences between the preoperative characteristics of the patients. No blood transfusions were required. Mean tumor size was 21.2 ± 7.0 mm. No patients demonstrated a delay in initiating oral intake or a regular diet. Mean hospital stay after surgery was 3.9 ± 1.0 days, and convalescence was complete at two weeks. The postoperative course was uneventful with no morbidity within one month of follow-up.

**Table 2:** Patient demographics

	**LESS adrenalectomy ( *n=14 *)**
Age (years) *	51.7 ± 11.6
Sex (Male/Female)	7/7
BMI (kg/m^2^) *	24.4 ± 3.3
Tumor location (Right/Left)	6/8
Previous abdominal operation	4
Indication for surgery	
Aldosterone-producing adenoma	11
Non-functioning tumor	2
Cushing’s syndrome	1

*Values are means ± standard deviation.

LESS; laparoendoscopic single site, BMI; body mass index.

**Table 3:** Operative outcomes

	**LESS adrenalectomy ( *n=14 *)**
Operating time (min) *	128.1 ± 31.5
Blood loss (ml) *	10.5 ± 12.1
Tumor size (mm) *	21.2 ± 7.0
Resumption of oral intake (days) *	1
Analgesics within 12 h after surgery (n)	2
Length of hospital stay (days) *	3.9 ± 1.0
Conversion to two port surgery	3
Morbidity	0
Mortality	0

*Values are means ± standard deviation.

LESS, laparoendoscopic single site.

**Figure 2:**
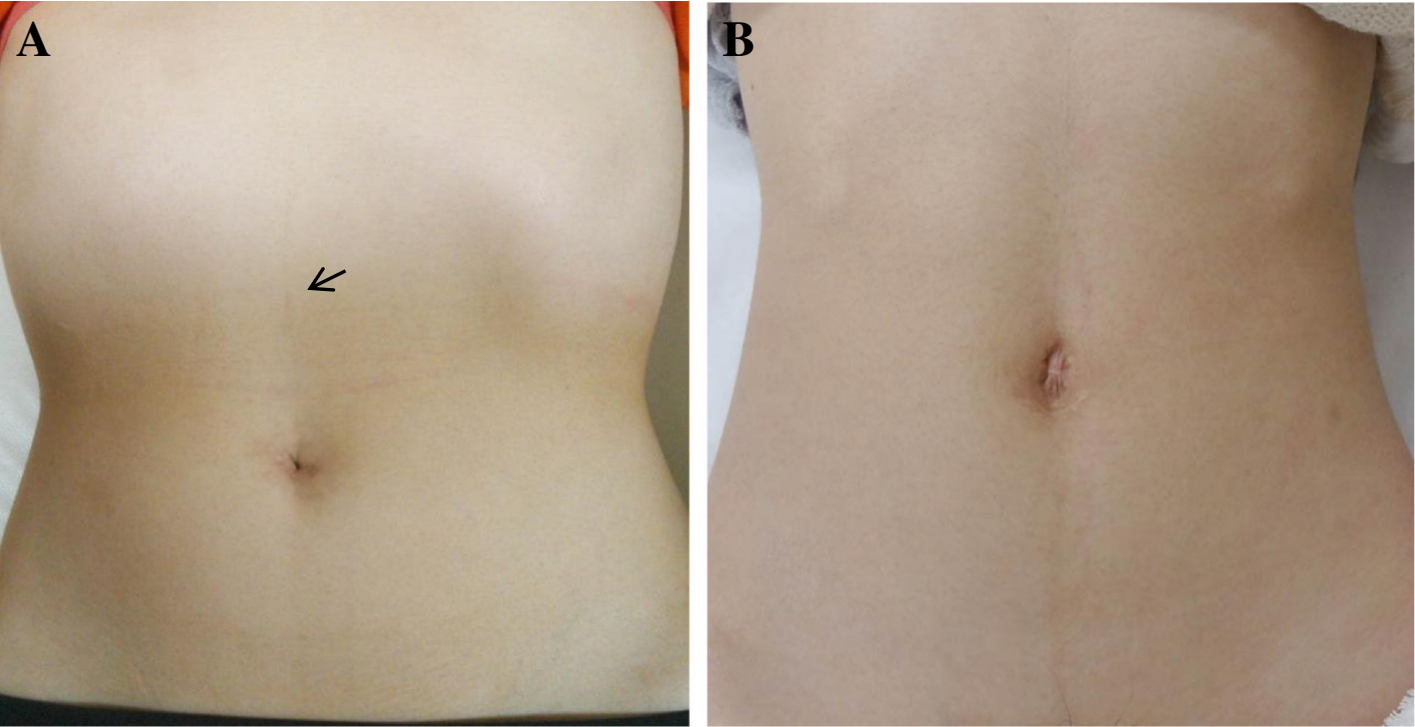
**Cosmetic results. A**: Postoperative photograph of patient’s abdomen at 8 months after LESS right adrenalectomy. *Black arrow* in the epigastric region points to the puncture site from the 2.3 mm needlescopic instrument. **B**: Postoperative photograph of patient’s abdomen at 3 years after LESS left adrenalectomy.

Mean scores for postoperative incisional pain on postoperative days 1, 3, and 14 were 2.3 ± 1.6, 1.0 ± 1.1, and 0.3 ± 0.7 points, respectively. The rate of analgesic use (diclofenac sodium suppository; 50 mg) was also lower within 12 hours after surgery (14%). According to their self-assessments, mean cosmesis scores on postoperative days 3 and 14 were 9.4 ± 0.7 and 9.8 ± 0.3 points, respectively. The rates of best cosmetic satisfaction (VAS score of 10) at 3 and 14 days were 57% and 79%, respectively. The patient has had an excellent cosmetic result on postoperative follow-up (Figure 2).

A limited cost analysis was performed on the series of patients undergoing SPLA. LESS adrenalectomy was associated with 18% lower mean operative charges compared with the standard MPLA (¥147,000 vs ¥180,000).

## Discussion

Conventional multiport laparoscopic surgery is the gold standard operative treatment for a variety of diseases. Generally, the goal has been to minimize the invasiveness of this procedure by reducing the number or size of the operating ports. Recently, LESS surgery was developed as an extension of standard laparoscopic minimally invasive procedures. The potential for decreased pain, faster recovery, and improved cosmesis has surgeons, their patients, and the industry interested in pushing the technique forward. In 2008, Castellucci et al. [6] reported the first LESS adrenalectomy in a 63-year-old female patient with a 4.5-cm left adrenal incidentaloma. They used a 3-port technique, introduced through a 2-cm supraumbilical incision and successfully removed a pheochromocytoma. However, LESS adrenalectomy is still limited by the surgical team’s adrenal and laparoscopic experience [6-8,12].

Since March 2009, we have been using LESS cholecystectomies in selected patients with benign gallbladder diseases. Additionally, our team has recently performed successful advanced LESS procedures, such as gastrectomy, colectomy, splenectomy, Heller-Dor procedure, and Nissen fundoplication [9-13]. At our institution, LESS adrenalectomy was introduced after more than 100 MPLAs were conducted. Since our first description in 2010 [12], we have performed LESS adrenalectomies on consecutive patients with benign adrenal tumors. In a LESS left adrenalectomy, as in conventional MPLA, the spleen is mobilized which provides a good operative field surrounding the left adrenal gland. The applicability of a LESS right adrenalectomy has not resulted in its widespread use, however, due to its technical challenges. The most important technical challenge for LESS right adrenalectomy is providing a good operative field surrounding the right-sided tumors. However, an elevation of the right liver lobe using a percutaneous instrument provided good visualization of the operative field, which reproduced a result similar to that observed in MPLA. The assistance of the needlescopic instrument does not compromise the cosmetic outcomes; this fact is considered to be one of the main advantages of LESS adrenalectomy over MPLA. An additional 5-mm port was required in two patients with body mass indexes greater than 27 kg/m^2^. Good laparoscopic skills and careful patient selection are essential; additional ports should be considered to help with liver retraction.

Two matched-control studies have reported that patients undergoing LESS adrenalectomy had significantly lower pain scores or required significantly less analgesia [7,14]. Jeong et al. [7] reported the first matched case–control study to demonstrate the technical feasibility of LESS adrenalectomy, compared with conventional MPLA, in the removal of a benign adenoma. Nine patients undergoing LESS adrenalectomies were compared with 17 patients undergoing conventional MPLA. No significant differences were found between the groups in terms of mean operating time, blood loss, or postoperative hospitalization. However, the degree of postoperative pain was significantly lower in the LESS adrenalectomy group than in the MPLA group. Our study also demonstrates that the postoperative VAS scores for incisional pain were lower. However, evaluation of postoperative cosmetic outcomes is a challenge, due to the absence of a reliable objective scale. The combination of multiple contributing factors, potential observer bias, and variations in patients’ expectations contributes to difficulties in assessing cosmetic outcomes [15]. In our series, we observed that patients scored the single-wound technique significantly better with regard to cosmetic appearance. However, operating surgeons should consider carefully which patients would be ideal candidates for initial LESS adrenalectomies.

## Conclusions

LESS adrenalectomy is a safe and technically feasible procedure for patients with benign adrenal tumors, and offers cosmetic benefit and the potential for postoperative pain reduction. However, surgeons with lack of experience as LESS surgery should be comprehended that the assistance of the needlescopic instrument does not compromise the cosmetic outcomes for difficult cases and the obese patients may not always be suitable candidates for pure LESS technique. Further studies are necessary to clearly identify the risks and benefits of this new approach to the adrenalectomy.

## Consent

Written informed consent was obtained from the patient for publication of this report and any accompanying images. A copy of the written consent is available for review by the Editor-in-Chief of this journal.

## Abbreviations

LESS: Laparoendoscopic single site; MPLA: Multiport laparoscopic adrenalectomy; VAS: Visual analog scale; SD: Standard deviations.

## Competing interest

The authors declare that they have no competing interests.

## Authors’ contributions

AS conceived the experimental plan, analyzed the data, and drafted the manuscript. NH, OK, SN, KK, MM, and GW helped to draft the manuscript. SB and AU cared for the patients. All authors read and approved the final manuscript.

## Pre-publication history

The pre-publication history for this paper can be accessed here:

http://www.biomedcentral.com/1471-2490/13/21/prepub
